# The effect of general practitioners’ sex and age on patients’ healthcare utilization: a Norwegian registry study

**DOI:** 10.1007/s43999-026-00086-4

**Published:** 2026-02-06

**Authors:** Schyler Marie Bennett, Kjartan Sarheim Anthun, Ottar Bjerkeset, Geir Godager, Johan Håkon Bjørngaard, Christina Hansen Edwards

**Affiliations:** 1 Department of Public Health and Nursing, Faculty of Medicine and Health Sciences, NTNU – Norwegian University of Science and Technology, Trondheim, Norway; 2 Department of Health Research, SINTEF Digital, Trondheim, Norway; 3 Faculty of Nursing and Health Sciences, Nord University, Levanger, Norway; 4 Institute of Health and Society, Department of Health Management and Health Economics, University of Oslo, Oslo, Norway; 5 Health Services Research Unit, Akershus University Hospital, Oslo, Norway; 6 Division of Mental Health Care, St. Olavs Hospital HF, Trondheim University Hospital, Trondheim, Norway; 7 Department of Child and Adolescent Health Promotion Services, Norwegian Institute of Public Health, Levanger, Norway

**Keywords:** General practitioners, Healthcare utilization, GP characteristics, Mental health services, Specialist care, Out-of-hours care.

## Abstract

**Purpose:**

This study aims to estimate the associations of general practitioner (GP) sex and age with patients’ use of specialist and out-of-hours healthcare services in Norway, using a quasi-experimental design that leverages patient assignment to a GP to reduce bias from patient-GP matching.

**Methods:**

Using national registry data on 1,884,665 adult patients assigned to a GP from 2008 to 2021, we exploited quasi-random assignment of patients to GPs to estimate the associations of the assigned GP’s sex or age with patient healthcare utilization in the three years after assignment. Poisson regression analyses were used for binary outcomes (contact/no contact), and linear regressions for continuous measures (number of contact days) of healthcare utilization, each with multi-level fixed effects to adjust for confounding. Analyses were stratified by patient groups defined by history of mental health diagnosis.

**Results:**

GP sex and age generally had small or null associations with healthcare utilization. A small negative association was observed for non-acute outpatient mental and somatic healthcare, but this was not consistent across groups defined by mental health diagnostic history. GP sex or age showed no consistent associations with other outcomes.

**Conclusion:**

As the GP workforce undergoes demographic changes—with an increasing share of younger and female physicians—these results suggest that such characteristics are unlikely to be strongly associated with individual patients’ use of specialist or out-of-hours mental and somatic healthcare services.

**Supplementary Information:**

The online version contains supplementary material available at 10.1007/s43999-026-00086-4.

## Introduction

In recent decades, the general practitioner (GP) workforce has undergone notable demographic changes, with a rising proportion of female and older GPs [1]. At the same time, GPs are facing increasing pressures due to increased workloads and high turnover rates [2]. Mental health conditions play an increasingly large role in general practice. In Norway, GPs are the first point of contact for mental health problems and gate-keep specialist care. Approximately 11% of all GP contacts in Norway are related to mental health [3, 4], with an increasing number of patients seeking GP care for mental health problems over time [5]. Research indicates that mental health consultations are generally longer than somatic consultations [6] and leave GPs with a stronger sense of insufficient time [7], though they may not contribute to a greater total workload [8].

As the GP workforce shifts to younger and more female physicians, this demographic shift could influence patterns of healthcare use. Much of the existing literature has focused on referral rates. Findings in Norway suggests that female GPs have higher referral rates to specialist care than male GPs [9], and that younger GPs also have higher referral rates, particularly in out-of-hours settings [10, 11], but broader evidence on how GP characteristics shape practice style and treatment decisions is mixed [12–17]. Although referral rates represent one pathway through which GP characteristics may influence healthcare utilization, there are distinct factors that shape healthcare utilization after referral. Evidence on referral rates, therefore, does not clearly indicate the impact on overall healthcare utilization. However, existing evidence on the associations of GP sex and age with healthcare utilization is unclear [18, 19] and remains understudied.

This is likely because the relationship between GP characteristics and patient healthcare use is complex and confounded by several sources of bias. First, patients may choose their GP based on characteristics such as sex and age. Patients may feel more comfortable and understood when their GP shares similar demographic traits, such as sex or age [20–22], particularly in the context of mental healthcare [23, 24]. Research indicates that patients tend to select GPs more similar to themselves [20, 25, 26]. Secondly, there are systematic differences in the population and healthcare needs of patients on GPs’ patient lists. These intertwined factors make it hard to determine whether observed differences in healthcare use stem from the GP’s characteristics or underlying differences in patient case mix. Therefore, there is a need for research that can disentangle the influence of GP characteristics from confounding patient- and practice-level factors when examining healthcare utilization.

In this study, we aim to estimate the association of GP sex and age with patients’ use of specialist and out-of-hours mental healthcare using a quasi-experimental design. To reduce bias due to patient-GP matching, we utilize a population of patients who are assigned to a GP. Given the connection between mental and somatic health [27], we further aimed to assess the association of GP sex and age with somatic healthcare utilization.

## Methods

The study is set in Norway, which has a universal, state-funded healthcare system with low out-of-pocket costs. GPs handle non-urgent care and serve as gatekeepers to specialist services. Further details on the organization and funding of healthcare services in Norway are provided in the Supplementary Methods, Section A.

### Study design & population

We linked individual-level data from national registries and Statistics Norway using pseudonymized patient IDs. Specialist healthcare data came from the Norwegian Patient Registry, GP and out-of-hours healthcare data from the Norwegian Control and Payment of Health Reimbursements Database, demographics from Statistics Norway, mortality data from the Cause of Death Registry, and GP characteristics and IDs from the Norwegian General Practitioner Registry.

#### GP assignment process

Nearly all Norwegian residents are enlisted with a GP [28], either by choice or through assignment. Assignment occurs when a GP reduces or closes their list, or when individuals immigrate to Norway. In these cases, a computer system randomly allocates patients to a GP with available list space in their municipality of residence [29]. In some cases of GP list reduction or closure, particularly in municipalities with a small number of GPs, the municipality may hire a GP to take over the patient list. Even in these cases, assignments are independent of the preferences of the patient, their previous GP, or the new GP [29]. We focus on patients who were assigned to a GP, exploiting this process to isolate the association of GP characteristics with healthcare use, consistent with established approaches [19, 30].

#### Study population

**Fig. 1:**
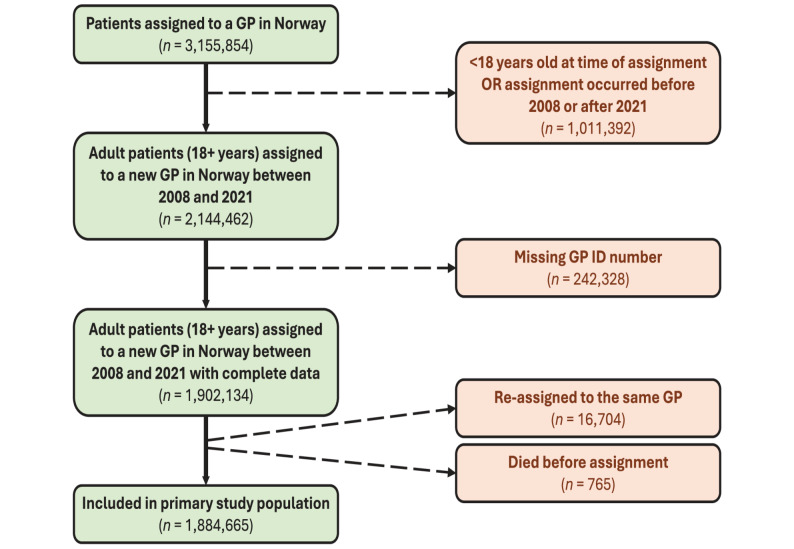
Flowchart of patient selection for inclusion in the primary study population. Green rectangles represent patients retained at each step; orange rectangles show exclusion criteria and number of excluded patients. Solid arrows indicate inclusion paths; dashed arrows indicate exclusion paths

Our primary study population included adults (18 + years of age) assigned to a GP between 2008 and 2021. We included only the first assignment per patient within the study period and set the assignment date as the index date. Patients who died before the index date or were re-assigned to the same GP (e.g. due to the GP relocating within a municipality) were excluded. Our final panel dataset included data on 1,884,665 patients’ use of specialist healthcare and out-of-hours services from January 1, 2008, to December 31, 2021. A flow chart of the study population selection process can be seen in Fig. 1.

### Study variables and definitions

#### GP characteristics

Through a unique pseudonymized GP ID, we retrieved information about the municipality the GP was working in and key characteristics of the GP. We used information about the GP’s sex (female as reference) and age (in years, at the index date). To capture the impact of shifts in the average age composition of GPs, GP age was modeled as a continuous variable, scaled for 10-year increases by dividing by a value of 10.

#### Patient characteristics

We included the following variables on patient demographics: education level (any education above high school level/no education above high school level achieved by October of year of assignment), father’s and mother’s education level (any education above high school level/no education above high school level in the year patient turned 16 years old), country of birth (born in Norway to Norwegian or foreign parents, or born outside of Norway to Norwegian or foreign parents), sex (female as reference group), and age (continuous in years).

To examine whether the associations of GP sex and age with healthcare utilization differed based on patients’ mental health history, we identified prior mental health problems using past healthcare contacts. Patients were classified as having past mental health problems if they had at least one GP contact with an International Classification of Primary Care 2 (ICPC-2) P-diagnosis, or at least one specialist care contact with an International Classification of Diseases Version 10 (ICD-10) F-diagnosis before assignment (or since January 1, 2008). We then created five categories concerning patients’ mental health history: (1) all assigned patients, regardless of past diagnosis; (2) patients with recurrent mental health problems, having a mental health diagnosis in all three years before assignment; and (3–5) patients with a diagnosis three, two, or one year before assignment, respectively. Only new mental health diagnoses were counted in categories (4) and (5)—meaning category (4) excluded patients from category (3), and category (5) excluded patients from categories (3) and (4).

#### Healthcare utilization

All healthcare utilization variables were defined as (1) contact: binary variable of contact/no contact in the given year, and (2) number of contact days: continuous variable of the number of days of contact in the given year. Healthcare contact included both physical- and e-consultations and was estimated for four different categories of care (acute inpatient, non-acute inpatient, non-acute outpatient care and out-of-hours care), each of which was divided between psychiatric (mental) and somatic care. Further details on the definitions of these variables can be found in the Supplementary Methods, Section B.

### Analyses

We analyzed the association between GP sex and age and having a healthcare contact using Poisson pseudo-maximum likelihood regressions, and the number of contact days using linear regressions. Estimates for having a contact are reported as the relative risk (specifically, the rate ratio) and estimates for contact days are reported as the change in the number of contact days. Poisson models followed patients each year until death or end of dataset, whichever came first. For the linear models, we excluded observations with less than 365 days until the end of the dataset, which effectively removed any observations where patients do not contribute a full year to the dataset. Both models included fixed effects and multi-way clustering on relevant variables (see Eq. 1, below). Analyses were conducted in Stata 18 [31] using the *ppmlhdfe* [32, 33] and *reghdfe* packages [34]. Plots were created in R [35], v.4.3.1, using *tidyverse* [36], *dplyr* [37], and *ggplot2* [38].

#### Primary analysis

In the primary analysis, we aimed to estimate the associations of the assigned GP’s sex and age with patients’ use of mental and somatic healthcare using the following equation:

1 $$\:\begin{array}{c}{HC\_use}_{imt}=\:{\beta\:}_{1}{GP\_char}_{imt}+{Y}_{t}+{ASP}_{im}+{\epsilon\:}_{imt}\:\end{array}$$

Where $\:{HC\_use}_{imt}$ represents one of eight healthcare outcomes (acute inpatient mental or somatic, non-acute inpatient mental or somatic, non-acute outpatient mental or somatic, and out-of-hours mental or somatic healthcare) assessed for individual $\:i$ in municipality $\:m$ at time $\:t$.

The independent variable is $\:{GP\_char}_{imt}$, the characteristic of the GP that individual $\:i$ was assigned to in municipality $\:m$ at time $\:t$, defined as either GP sex (0 = female, 1 = male) or GP age (scaled for each 10-year increase).

The term $\:{\beta\:}_{1}$ represents the estimation coefficient.

We included several fixed effects to analyze only within-group variability, allowing for control of confounding factors which are constant in groups, similar to a previous study [10]. In total, the model included four fixed effect variables, split up into two terms. The first fixed effect term, $\:{Y}_{t}$, consists of one variable for the calendar year of assignment date to capture time-specific variation that may influence healthcare utilization. The second fixed effect term, $\:{ASP}_{im}$, consists of three fixed effect variables: patient age (age, in years, of individual $\:i$), patient sex (defined as 0 for female and 1 for male), and the patient’s previous GP (defined as the ID of the previous GP of individual $\:i$). We include the previous GP as a fixed effect to account for systematic differences between GP lists, particularly in cases where a large proportion of patients are transferred to the same new GP. Taken together, we analyze the within-group variability for patients within *(i)* each year and *(ii)* assigned from the same GP with the same age and sex.

Standard errors were clustered at the municipality, GP, and individual levels to account for within-municipality correlation in the error term ($\:{\epsilon\:}_{imt}$), as well as potential clustering at the GP and individual levels, thus addressing potential heteroskedasticity and spatial dependence.

#### Stratification of the primary analysis

We stratified by patients’ mental health history, using the groups described previously, as healthcare needs are likely to vary between subgroups. We also stratified the analysis by patient sex to assess whether the associations of GP sex and age differed between male and female patients.

#### Assessment of the assignment process: balance tests

To assess whether the GP assignment process was unrelated to patient characteristics, we conducted a series of balance tests. These tests assessed associations between patient characteristics and the sex or age of the assigned GP. Further methodological details and equations (Equations S1 – S3) are provided in the Supplementary Methods, Section C.

#### Healthcare utilization before assignment

As a sensitivity analysis, we also examined patients’ healthcare use in the two years *prior* to assignment. We expected that the sex and/or age of the assigned GP should be unrelated to the health care utilization of the patient prior to assignment.

#### Post-assignment GP switching

The primary analysis included all patients who were assigned to a new GP, regardless of compliance with the assignment, similar to an intention-to-treat analysis. As a sensitivity analysis, we stratified the analyses by the categories of the switch after assignment variable. This variable had the following categories: (1) no further switches, (2) patient-initiated switch in the first year after assignment, (3) patient-initiated switch in the second year after assignment, (4) patient-initiated switch in the third year after assignment, and (5) GP-initiated switch any time after assignment. Here, a patient-initiated switch occurs when a patient chooses a new GP, and a GP-initiated switch occurs when a GP reduces or closes their list.

#### Robustness check comparing analytical approaches and study populations

Our study focuses on a unique subpopulation of the Norwegian population—patients assigned to a GP—allowing us to apply a quasi-experimental design. To parallel the main approaches used in prior studies—covariate-adjusted regression in a population which could select a GP [18] and difference-in-difference methods in a GP assignment setting [19]—we conducted a robustness check to assess how the findings might differ under alternative analytical approaches and definitions of the study population. Specifically, we compared results from our primary analysis—which accounts for multi-level fixed effects and leverages the population of assigned patients to minimized confounding from patient-GP matching—to results based on a more conventional model, which included covariates and a single fixed effect. This more conventional model was applied to both the population of assigned patients and in a broader population that included patients who have selected their GP.

First, we modified our regression model to include covariates of patient characteristics while maintaining year fixed effect and standard error clustering as in the primary analysis (Equation S4, Supplementary Methods, Section D). As mentioned above, this equation was applied to (1) our primary study population of all adult patients assigned to a GP and (2) a broader population including all adult Norwegian residents from 2008 to 2021. Patients in this broader population were followed from either the start of the dataset (January 1st, 2008), the year they turned 18, or the year they entered the GP scheme, until emigration or withdrawal from the GP scheme, death, or the end of the dataset (December 31st, 2021), whichever came first. Since the broader population was not tied to a GP assignment date, a patient’s GP was defined as the one they were registered with on January 1st of each year. The results from this regression can therefore be interpreted as the average associations of GP sex and age with patient utilization of healthcare from 2008 to 2021 for all adult residents in Norway.

## Results

The study population included 1,884,665 patients assigned to a new GP from 2008 to 2021, each followed for three years after assignment. About half (51%) were female, and the mean age was 47 years. Approximately 7% of patients had a recurrent mental health diagnosis in the data period. Patients were assigned to 7,178 unique GPs, of whom 43% were female, with a mean age of 44 years. Full descriptive statistics for patients can be found in Table 1 and for GPs in Table 2.

**Table 1:** Descriptive table of characteristics of study population

Patient characteristics		Mean		Standard deviation
**Age**		47.35		19.09356
		**Percent (%)**		**N**
**Sex**	Male	49.21	927,471	
	Female	50.79	957,194	
**Country of birth**	Norway	81.51	1,536,244	
	Outside Norway	18.49	348,417	
	Missing	00.00	4	
**Education** ^**a**^	No higher education	37.73	711,127	
	Any higher education	58.65	1,105,283	
	Missing	3.62	68,255	
**Mother’s education** ^**b**^	No higher education	50.85	958,400	
	Any higher education	21.20	399,592	
	Missing	27.95	526,673	
**Father’s education** ^**b**^	No higher education	40.55	764,258	
	Any higher education	28.83	543,423	
	Missing	30.61	576,984	
**Reason for assignment**	Previous GP closed or reduced list	97.32	1,834,229	
	Immigration to Norway	2.68	50,436	
**Mental health diagnosis**^**c**^ **history**	All patients	100.00	1,884,665	
	Recurrent mental health problem ^**d**^	7.03	132,408	
	Mental health diagnosis 3 years before assignment	14.65	276,148	
	Mental health diagnosis 2 years before assignment ^**e**^	6.60	124,345	
	Mental health diagnosis 1 year before assignment ^**f**^	5.26	99,111	

^a^Education defined as no education above high school level/ any education above high school level achieved by October of year of assignment

^b^Mother’s/father’s education defined as no education above high school level/ any education above high school level in the year patient turned 16 years old

^c^Mental health diagnosis defined as a P diagnosis from International Classification of Primary Care 2 (ICPC-2) and/or an F diagnosis from International Classification of Diseases Version 10 (ICD-10)

^d^Defined as having a mental health diagnosis in all three years before assignment

^e^Defined as a new mental health diagnosis 2 years before assignment, i.e. excluding those with a mental health diagnosis 3 years before assignment

^f^Defined as a new mental health diagnosis 1 year before assignment, i.e. excluding those with a mental health diagnosis 2 or 3 years before assignment

**Table 2:** Descriptive table of characteristics of the general practitioners (GPs) included in the study population

GP characteristics		Assigned GP		Previous GP	
		Mean	Standard deviation	Mean	Standard deviation
**Age**		43.54	12.26	49.20	12.38
		**Percent**	**N**	**Percent**	**N**
**Sex**	Male	42.56	3,055	39.77	2,640
	Female	57.44	4,123	60.23	3,998
**Total**		100.00	6,638	100.00	7,178

### Primary analysis

**Fig. 2:**
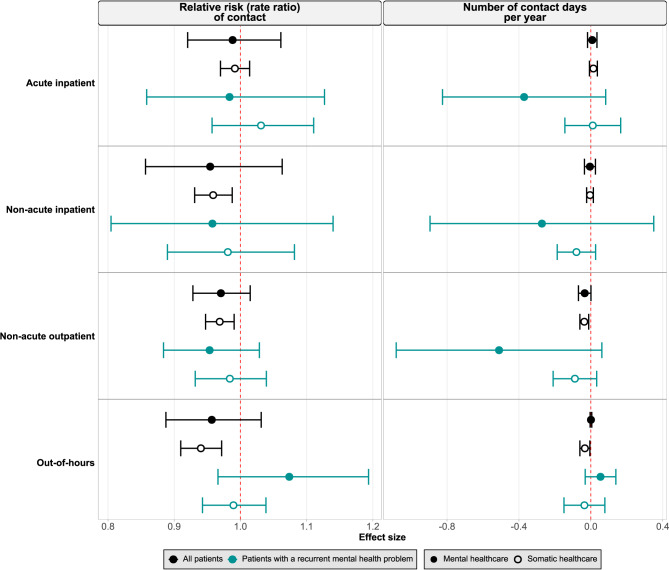
Estimated association of assignment to a male general practitioner (GP) with the relative risk of contact (left panel) and number of contact days per year (right panel) with specialist and out-of-hours healthcare. Each row represents a healthcare setting (acute inpatient, non-acute inpatient, non-acute outpatient, out-of-hours). Black symbols represent all patients; turquoise symbols represent patients with a recurrent mental health problem (defined as having a mental health diagnosis in all three years before assignment). The reference category was assignment to a female GP. Filled symbols indicate mental healthcare, and hollow symbols indicate somatic healthcare. Horizontal lines show 95% confidence intervals. All estimates are from the second year after assignment

**Fig. 3:**
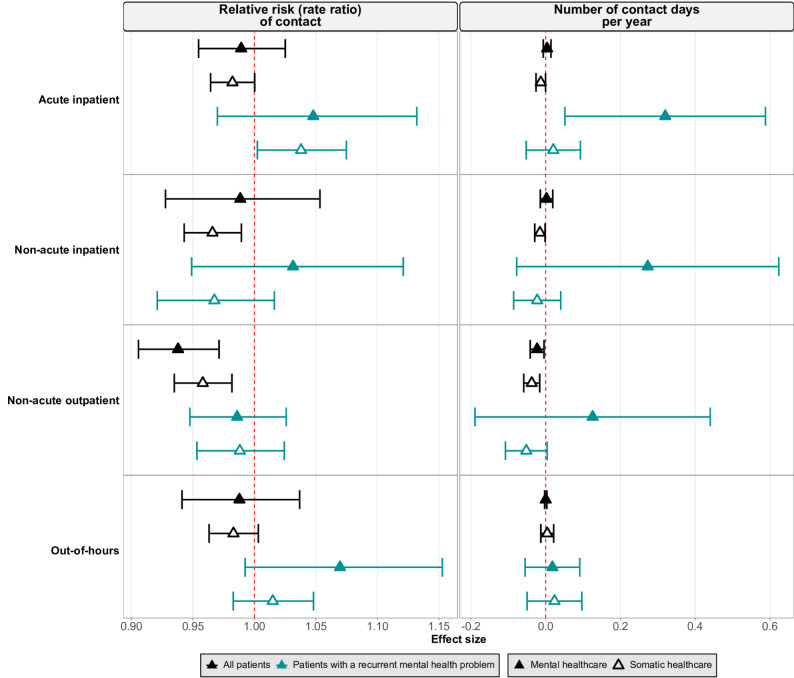
Estimated association of each 10-year increase in general practitioner (GP) age with the relative risk of contact (left panel) and number of contact days per year (right panel) with specialist and out-of-hours healthcare. Each row represents a healthcare setting (acute inpatient, non-acute inpatient, non-acute outpatient, out-of-hours). Black symbols represent all patients; turquoise symbols represent patients with a recurrent mental health problem (defined as having a mental health diagnosis in all three years before assignment). Filled symbols indicate mental healthcare, and hollow symbols indicate somatic healthcare. Horizontal lines show 95% confidence intervals. All estimates are from the second year after assignment

In this section, we report the estimated associations of GP sex (Fig. 2) and age (Fig. 3) with healthcare utilization in the second year after assignment amongst all assigned patients and patients with a recurrent mental health problem. The estimates for other years and patient groups can be seen in Fig. S1 – S4 in the Supplementary Results. For GP sex, each result represents the estimated association between assignment to a male GP with the probability or number of contact days with mental or somatic specialist and out-of-hours healthcare, compared to the reference category of assignment to a female GP. For GP age, each result represents the estimated association between each 10-year increase in the assigned GP’s age with these same outcomes.

The association of GP sex with the relative risk of contact with specialist and out-of-hours healthcare was small or null. All patients assigned to a male GP had a slightly lower relative risk of contact with each type of care except acute inpatient healthcare, which was approximately null (Fig. 2). The association of GP age with the relative risk of contact with specialist and out-of-hours healthcare showed similar patterns, with each 10-year increase in GP age associated with a slightly lower relative risk of contact with each type of care (Fig. 3).

These findings represent the estimates for each individual patient. To demonstrate the potential impacts on the healthcare system, we report the percent change in the number of patients with specialist or out-of-hours healthcare contact in 2019 (the most recent pre-pandemic year) under a hypothetical scenario in which all patients were assigned to a male GP (Table S1, Supplementary Results). Given this scenario, the percentage of patients with contact with non-acute outpatient mental and somatic healthcare might have been modestly lower (approximately − 2.1% for mental healthcare and − 1.44% for somatic healthcare; Table S1, Supplementary Results).

The associations of GP sex and age with the number of contact days per year were also small (Figs. 2 and 3). For both exposures, estimates for somatic and mental healthcare generally agreed in direction, though the CIs for mental healthcare were wider than those for somatic.

#### Stratification of the primary analysis

The results amongst patients with a recurrent mental health problem did not substantially deviate from the results for all assigned patients. A few estimates were opposite in direction between all assigned patients and patients with a recurrent mental health problem, but the CIs for the latter group were wider (Figs. 2 and 3).

The sex-stratified analyses showed no substantial differences between male and female patients (Fig. S3–S4 in Supplementary Results). The associations of GP sex with the relative risk of acute inpatient and non-acute outpatient mental and somatic healthcare were opposite in direction between male and female patients, though this was not consistent between patient groups (Fig. S3 in Supplementary Results).

### Assessment of the assignment process: balance tests

GP sex showed little to no association with patient characteristics, except for a small association with patient sex. GP age showed small associations with patient characteristics, including patient age, country of birth, education, and parental education (both father’s and mother’s). Details can be seen in Fig. S5, Supplementary Results.

### Healthcare utilization before assignment

GP sex and age had little association with specialist and out-of-hours healthcare utilization in the two years prior to assignment (Fig. S6 – S7, Supplementary Results). While GP sex and age showed some associations with specific types of care, the effect sizes were small and did not suggest a broader pattern.

### Post-assignment GP switching

Overall, there was little to no association of GP sex or age with specialist or out-of-hours healthcare utilization amongst patients who did not switch after re-assignment and those who did switch at different time points (Fig. S8 – S9, Supplementary Results).

### Robustness check comparing analytical approaches and study populations

**Fig. 4:**
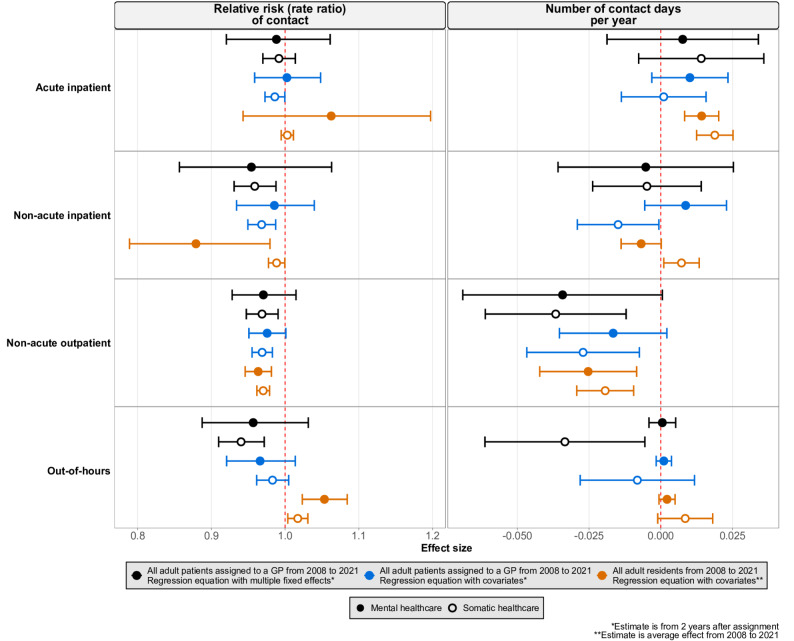
Estimated association of general practitioner (GP) sex with the relative risk of contact (left panel) and number of days per year (right panel) of contact with specialist and out-of-hours healthcare. Each row represents a healthcare setting (acute inpatient, non-acute inpatient, non-acute outpatient, out-of-hours). Black symbols represent the study population of all adult patients assigned to a GP, estimated using a regression model with multiple fixed effects (Equation 1); blue symbols represent the same study population, estimated with a modified regression model including covariates (Equation S4, Section D, Supplementary Methods); orange symbols represent a broader study population of all adult residents (2008 – 2021), estimated with the modified regression model including covariates (Equation S4, Section D, Supplementary Methods). The reference category was assignment to a female GP (black and blue symbols) or being registered with a female GP (orange symbols). Filled symbols indicate mental healthcare, and hollow symbols indicate somatic healthcare. Horizontal lines show 95% confidence intervals. Estimates for the primary study population (black and blue symbols) reflect the second year after assignment; estimates for the broader population (orange symbols) are averaged across 2008–2021

**Fig. 5:**
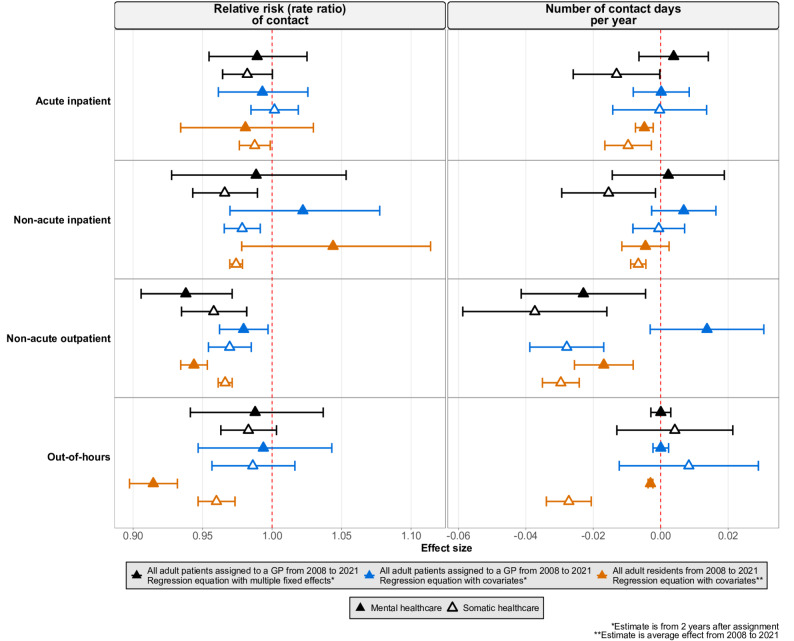
Estimated association of **e**ach 10-year increase in the age of the general practitioner (GP) with the relative risk of contact (left panel) and number of contact days per year (right panel) with specialist and out-of-hours healthcare. Each row represents a healthcare setting (acute inpatient, non-acute inpatient, non-acute outpatient, out-of-hours). Black symbols represent the study population of all adult patients assigned to a GP, estimated using a regression model with multiple fixed effects (Equation 1); blue symbols represent the same study population, estimated with a modified regression model including covariates (Equation S4, Section D, Supplementary Methods); orange symbols represent a broader study population of all adult residents (2008 – 2021), estimated with the modified regression model including covariates (Equation S4, Section D, Supplementary Methods). Filled symbols indicate mental healthcare, and hollow symbols indicate somatic healthcare. Horizontal lines show 95% confidence intervals. Estimates for the primary study population (black and blue symbols) reflect the second year after assignment; estimates for the broader population (orange symbols) are averaged across 2008–2021

In our robustness check, in which we applied a modified version of our regression equation to both our primary study population (all adult patients assigned to a GP between 2008 and 2021) and to a broader population of all adult residents in Norway during the same time period, the estimates for the association of GP sex (Fig. 4) and age (Fig. 5) with healthcare utilization remained small or null. Estimates for out-of-hours healthcare utilization in the broader population differed slightly across analyses, though the differences were small.

## Discussion

This study aimed to estimate the associations of GP sex and age with patients’ use of specialist and out-of-hours healthcare for mental and somatic conditions. The associations of GP sex and age with healthcare utilization were consistently small or null in this study. We found that being assigned to a male GP and each 10-year increase in GP age had a small negative association with patients’ healthcare utilization across most categories of healthcare. Using a hypothetical scenario in which all patients were instead assigned to a male GP, we show that the projected impact of these estimates on the healthcare system would be small.

Further, although GP sex and age had little association with healthcare utilization amongst all assigned patients, the estimate could differ across patient populations. We therefore stratified by the timing and duration of mental health diagnoses and by patient sex, and found consistent estimates across groups. Although men and women have different utilization rates [18, 39], our findings align with another study which found no association between GP-patient sex concordance and specialist mental healthcare utilization [18].

The robustness check applied a modified version of our regression equation to both our primary study population and to a broader population of all adult residents in Norway. This allows for comparison of our quasi-experimental design—which includes multi-level fixed effects and a population of assigned patients—with existing analytical approaches and study populations which include self-selected patient-GP pairs. The estimates from the robustness check should be interpreted with greater caution, as replacing fixed effects with covariate adjustment and including unassigned patients likely introduced additional sources of confounding and reduced internal validity. Although some estimates in the broader study population differed in size or direction from those for assigned patients, suggesting potential under- or over-estimation, the estimates in this analysis were generally small or null. Together with prior studies, this suggests that the estimated associations are robust across methods and population.

Prior studies have generally found no association between GP sex or age and patient healthcare utilization, similar to our findings. A study conducted in Norway found that GP sex and age had no association with patients’ likelihood of specialist mental healthcare utilization, though their study population included patients who had chosen their GP [18]. These findings are consistent with broader evidence that specialist mental healthcare contacts in Norway remained relatively stable between 2010 and 2020 [5]. Another study conducted in Austria, which included only patients assigned to a GP, found that GP sex had no association with patients’ specialist healthcare utilization, though there is no strict gatekeeping system in Austria, amongst other differences [19].

Much of the existing literature has focused on the relationship between GP characteristics and referral rates. Though some research suggests that female [9] and younger GPs [10, 11] may have higher referral rates than male and older GPs, particularly in out-of-hours settings, the overall evidence remains mixed [13–17]. Importantly, although referral decisions are complex [40], there are distinct factors that shape healthcare utilization after referral. First, approximately 25% of referrals to specialist mental healthcare services for adults are rejected in Norway [41]. Second, even if they are accepted, patient characteristics such as country of birth [42], frequency of GP contacts [18], sex [18, 39], and socio-economic status [43–45] may influence use of specialist mental healthcare. Third, as not all referrals come from GPs, particularly in acute healthcare services [46], GP-related effects on referral rates may not be equally relevant across different types of care. These factors might explain why studies on healthcare utilization, including ours, find small or no associations, whereas some studies on referral rates report associations.

### Strengths & Limitations

A key strength of this study is the use of individual-level registry data, covering nearly the entire population of Norway over an extended period. Moreover, our quasi-experimental design, leveraging GP assignment and fixed effects, limited selection bias and addressed temporal and contextual variation. Our approach aligns with established designs that leverage GP assignments [19, 30] and multi-level fixed effects [10] to quantify associations.

Although GP assignment was not based on patient choice, our balance tests provide some evidence of association with patient characteristics. This suggests that the GP assignment process may involve a degree of non-random allocation. Such imbalances may arise from organizational or location-specific factors. For example, in small municipalities, closed or reduced lists are often transferred to a single incoming GP. If the likelihood of taking these positions varies by GP sex or age, these characteristics may cluster systematically, creating aggregate imbalance. Nevertheless, the associations between GP sex and GP age and patient characteristics were small, making it unlikely that any residual confounding would fully mask substantial underlying associations.

Several elements outside the scope of the current study present opportunities for future research. First, Z-diagnoses, ICPC-2 codes which describe social problems and stressors, may be used alongside or instead of P-diagnoses, and their use in characterizing mental health problems could be explored. Second, although private for-profit care use in Norway is quite low [47], its inclusion could provide additional insights on healthcare utilization. Third, examining substance use disorders separately may reveal distinct patterns of health needs and utilization. Fourth, although GP specialization and practice type variables were available, they were not pursued due to limited variation; these factors could be explored in other settings. Fifth, we did not investigate the interaction of GP sex and age or potential non-linear effects of GP age; both could be examined further. Finally, our study did not assess the overall magnitude of variation due to GP characteristics or other factors. Future studies could examine these and broader sources of variation, as prior research indicates that GPs vary in their mental health-related expertise and interest [48–51], patient list characteristics [52], and service organization and reimbursement [53].

Additionally, although our study accounts for case mix differences between municipalities and over time, it was not designed to determine the quality or necessity of care. Our results should therefore be interpreted as differences in healthcare utilization, and further research is needed to clarify whether these differences correspond to the quality or necessity of care.

## Conclusion

The estimates for GP sex and age found in this study suggest that these characteristics have a small association with healthcare utilization in our study population, with patterns remaining similar regardless of whether patients were assigned to younger or older, male or female GPs. As the GP workforce undergoes demographic changes—with an increasing share of younger and female physicians—these results suggest that such characteristics are unlikely to be strongly associated with individual patients’ use of specialist or out-of-hours mental and somatic healthcare services.

## Supplementary Information

Below is the link to the electronic supplementary material.

Supplementary Material 1

Supplementary Material 2

## Data Availability

This study is based on the use of clinical and administrative registry data. Therefore, the data underlying this study cannot be shared publicly due to legal restrictions.

## Code availability

Code used to conduct the analysis and to create the figures can be shared upon request.
