# Supplementary material for: The effect of general practitioners’ sex and age on patients’ healthcare utilization: a Norwegian registry study
Source: Res Health Serv Reg. 2026 Feb 6;5:4. doi: 10.1007/s43999-026-00086-4 (PMC12881246; doi:10.1007/s43999-026-00086-4)
Supplement: Supplementary file 1 — Supplementary Material 1 [file 43999_2026_86_MOESM1_ESM.docx]

Supplementary Methods

**Bennett SM***, Anthun KS, Bjerkeset O, Godager G, Bjørngaard JH, Edwards CH. *The effect of general practitioners’ sex and age on patients’ healthcare utilization: a Norwegian registry study.* Research in Health Services & Regions.

***Corresponding author:**

Schyler Marie Bennett | schyler.bennett@ntnu.no | Department of Public Health and Nursing, Faculty of Medicine and Health Sciences, NTNU – Norwegian University of Science and Technology; Trondheim, Norway

Table of Contents

Section A: Organization & funding of healthcare services in Norway 2

Section B: Definitions of variables for healthcare utilization 2

Section C: Assessment of the assignment process: Balance tests 4

Section D: Robustness check comparing analytical approaches and study populations 5

References 8

## Section A: Organization & funding of healthcare services in Norway

A national regular GP scheme was introduced in Norway in 2001. The scheme is intended to ensure that all residents in Norway have the possibility to enroll with a specific regular GP. Enrollment in the scheme has been high, with more than 99% of the population in Norway between 2008 and 2020 participating [1]. The municipalities are responsible for organizing the scheme and for ensuring that residents have access to a regular GP. Most patients have a GP that is affiliated with the municipality in which they reside.

The majority of GPs are self-employed and remunerated by the municipality through a combination of a capitation fee determined by the number of patients on the GP’s list, a fee-for-service made up of activity-based claims registered at the end of each consultation, and a patient copayment. A smaller, yet increasing, number of GPs are directly contracted by the municipality and have a fixed salary, but these GPs are still required to submit tariff-based reimbursement claims, with the municipality retaining this fee-for-service income—though not always for out-of-hours services.

Out-of-hours services in Norway are also organized by the municipalities. Some municipalities organize the service as a GP cooperative, in which GPs and other physicians work a certain number of hours at the service in addition to their other duties, whereas others are serviced entirely by physicians working only at out-of-hours services. Thus, the roles for GPs in the out-of-hours service varies widely between and even within municipalities, with GPs in small municipalities reporting the highest proportion of out-of-hours work within a week [2].

Both mental and somatic specialist healthcare services are funded by four regional health authorities. Services are located at hospitals, outpatient clinics, and at clinics of private specialists on contract with the regional health authorities.

Adult patients pay a small out-of-pocket copayment for GP and out-of-hours visits (about EUR 15 for daytime and about EUR 26 for evening/night visits in 2025) [3] and specialist healthcare consultations (about EUR 34) [4, 5]. If patients are admitted for inpatient care in hospitals, they do not pay a user fee. Total healthcare spending per patient is capped per year, with the limit for 2025 set at about EUR 282 [6].

## Section B: Definitions of variables for healthcare utilization

**Definition of acute and non-acute**

The acuteness of care is defined based on the degree of urgency registered by the specialist healthcare provider upon patient arrival. The degree of urgency is encoded by a variable called level of urgency, or *innmåte hastegrad* in Norwegian, in the Norwegian Patient Registry [7], where a value of 1 is considered to be acute care, and a value of 4 is considered non-acute care in this study.

**Definition of inpatient, outpatient, and out-of-hours**

Whether the care was inpatient or outpatient was defined based on a variable called service level, or *omsorgsnivå* in Norwegian, in the Norwegian Patient Registry [8]. A value of 1 corresponds to inpatient care and a value of 2 corresponds to day treatment, and these values were considered to be inpatient care in this study. A value of 3 corresponds to outpatient care and was thus used to define outpatient care variables in this study. Out-of-hours care was determined based on a practice reimbursement basis of either out-of-hours or municipal out-of-hours, *legevakt* and *kommunal legevakt* in Norwegian, in the Norwegian Control and Payment of Health Reimbursements Database. A value of 12 or 13 corresponds to out-of-hours services. All types of contact with out-of-hours services (physical consultations, e-consultations, telephone contact) were included.

**Definition of mental and somatic healthcare**

For specialist care outcomes (acute inpatient, non-acute inpatient, non-acute outpatient), the care was determined to be somatic if it was registered at a somatic healthcare service (e.g. hospital, somatic outpatient services), and psychiatric (mental) if it was registered at a psychiatric healthcare service (e.g. outpatient mental healthcare service). For out-of-hours outcomes, the care was determined to be mental health related if the contact had an attached P diagnosis, and somatic if there was no attached P diagnosis.

## Section C: Assessment of the assignment process: Balance tests

To assess if the GP assignment process is random (unrelated to patient characteristics), we conducted a series of balance tests. We evaluated whether patient characteristics were associated with the characteristics of the assigned GP. We evaluated the following patient variables: education (no education above high school level/any education above high school level in October of the year of assignment), father’s and mother’s education (no education above high school level/any education above high school level for the father and mother of individual $i$, in the year individual $i$ was 16 years old), country of birth (born in Norway to Norwegian or foreign parents, or born outside of Norway to Norwegian or foreign parents), sex (male/female, female as reference group), and age (years, scaled for 10-year increase [i.e. divided by 10]).

Each of these variables was assessed in regression analyses using the following equations:

$$\begin{aligned} {Patient\_char}_{{imt}_{1}}= \beta_{1}{GP\_char}_{imt}+Y_{t}+{AP}_{im}+\varepsilon_{imt} \#\left( S1 \right) \end{aligned}$$

$$\begin{aligned} {Patient\_char}_{{imt}_{2}}= \beta_{1}{GP\_char}_{imt}+Y_{t}+{SP}_{im}+\varepsilon_{imt} \#\left( S2 \right) \end{aligned}$$

$$\begin{aligned} {Patient\_char}_{{imt}_{3-6}}= \beta_{1}{GP\_char}_{imt}+Y_{t}+{ASP}_{im}+\varepsilon_{imt} \#\left( S3 \right) \end{aligned}$$

Where **Equation S1** is for the outcome variable of patient sex (${Patient\_char}_{{imt}_{1}}$), where the fixed effect for patient sex is therefore dropped; the fixed effect term ${AP}_{im}$ thus includes the variables patient age and previous GP ID. **Equation S2** is for the outcome variable of patient age (${Patient\_char}_{{imt}_{2}}$), where the fixed effect for patient age is therefore dropped; the fixed effect term ${SP}_{im}$ thus includes the variables patient sex and previous GP ID. **Equation S3** is for the outcome variables of patient country of birth, education, mother’s education, and father’s education (${Patient\_char}_{{imt}_{3-6}}$), where all fixed effects are present; the fixed effect term ${ASP}_{im}$ thus includes the variables of patient age, patient sex, and previous GP ID. Robust standard errors, $\varepsilon_{imt}$, were clustered by the ID numbers for individual $i$ and the GP of individual $i$, and by municipality.

## Section D: Robustness check comparing analytical approaches and study populations

To parallel the main approaches used in prior studies—covariate-adjusted regression in a population which could select a GP [9] and difference-in-difference methods in a GP assignment setting [10]—we conducted a robustness check to assess how the findings might differ under alternative analytical approaches and definitions of the study population. First, we created a modified regression equation (**Equation S4**). This equation includes covariates of patient characteristics that might influence both exposure and outcomes but keeps the same year-fixed effect and standard error clustering as in **Equation 1**.

$$\begin{aligned} {HC\_use}_{imt}= \beta_{0}+\beta_{1}{GP\_char}_{imt}+X_{imt}+Y_{t}+\varepsilon_{imt} \#\boldsymbol{(}S4\boldsymbol{)} \end{aligned}$$

**Equation S4** was applied in two study populations: ***(1)*** our primary study population of all adult patients assigned to a GP from 2008 to 2021 and ***(2)*** a broader population including all adult Norwegian residents from 2008 to 2021 (see the heading *Robustness check comparing analytical approaches and study populations* in the main text for further explanation).

Here, ${HC\_use}_{imt}$ represents one of eight types of healthcare utilization variables, as defined above. The independent variable is again ${GP\_char}_{imt}$, here defined as the characteristic of the GP that individual $i$ was assigned to in municipality $m$ at time $t$ for study population (1) or the characteristic of the GP that individual $i$ was registered with in municipality $m$ as of January 1^st^ of year $t$ for study population (2).

The term $X_{imt}$ is a vector of covariates of patient characteristics that may influence ${GP\_char}_{imt}$ and ${HC\_use}_{imt}$. The covariates are defined the same in study population (1) and (2) and include:

- Education level (no education above high school level/any education above high school level of individual $i$ in October of year $t$)
- Country of birth (born in Norway to Norwegian or foreign parents/born outside of Norway to Norwegian or foreign parents)
- Sex (male/female, female as reference category)
- Age (patient age in years, grouped by 5-year age categories)

Patient age was categorized in 5-year intervals in this model to better align with the categories created by the fixed effect in **Equation 1**, which effectively creates 1-year age categories for within-category comparisons.

The term $\beta_{0}$ represents the intercept.

The model includes only one fixed effect term, $Y_{t}$, a fixed effect for the calendar year of assignment in study population (1) or the calendar year of observation in study population (2).

Robust standard errors, represented by the term $\varepsilon_{imt}$, were clustered by the ID numbers for individual $i$ and the GP of individual $i$, and by municipality. The GP of individual $i$ was defined as the assigned GP in study population (1) or the GP that individual $i$ was registered with on January 1 of the given year in study population (2).

# References

1. Helsedirektoratet. Styringsdata for fastlegeordningen, 4. kvartal 2020 [Management data for the GP service, 4th quarter 2020] [Internet]. Oslo: Helsedirektoratet; 2020 [cited 2025 Jan 29]. Available from: https://www.helsedirektoratet.no/statistikk/fastlegestatistikk/Hovedtallsrapport%20fastlegeordningen%20landstall%202020-4.pdf/_/attachment/inline/987b5db5-9d42-48bf-badb-6f225eea825b:11c3859a805483c6183d8d4d1deaf2de66903ef8/Hovedtallsrapport%20fastlegeordningen%20landstall%202020-4.pdf.

2. Rebnord IK, Eikeland OJ, Hunskår S, Morken T. Fastlegers tidsbruk [General practitioner's time use]. Bergen: Nasjonalt kompetansesenter for legevaktmedisin, Uni Research Helse; 2018. Available from: https://www.helsedirektoratet.no/forebygging-diagnose-og-behandling/organisering-og-tjenestetilbud/fastlegeordningen-legevakt-og-andre-allmennlegetjenester/statistikk-og-rapporter-om-fastlegeordningen-legevakt-og-andre-allmennlegetjenester/_/attachment/inline/08f5d967-3d66-4701-ae3e-ab85f643726f:d93c603ae9befac348c2e29c341eb119f3f58990/Rapport%20Fastlegers%20tidsbruk%202018.pdf

3. Helfo. User fees at the family doctor. [Internet]. Oslo: The Norwegian Directorate of Health; 2024 [updated September 2, 2024 cited October 31, 2024]. Available from: https://www.helsenorge.no/en/payment-for-health-services/user-fees-at-the-family-doctor/.

4. Helfo. User fees and patient fees at hospitals and outpatient clinics. [Internet]. Oslo: The Norwegian Directorate of Health; 2024 [updated July 1, 2024; cited October 31, 2024]. Available from: https://www.helsenorge.no/en/payment-for-health-services/user-fees-at-hospitals-and-outpatient-clinics/.

5. Helfo. User fees for treatment by a psychologist. [Internet]. Oslo: The Norwegian Directorate of Health; 2024 [updated September 2, 2024; cited October 31, 2024]. Available from: https://www.helsenorge.no/en/payment-for-health-services/user-fees-for-treatment-by-a-psychologist/.

6. Helfo. Exemption card for public health services. [Internet]. Oslo: The Norwegian Directorate of Health; 2024 [updated September 2, 2024; cited October 31, 2024]. Available from: https://www.helsenorge.no/en/payment-for-health-services/exemption-card-for-public-health-services/.

7. Helsedataservice [Health Data Service]. Innmåte hastegrad [Level of urgency]. [Internet]. 2025 [cited 2025 May 13]. Available from: https://helsedata.no/no/variabler/?page=search&variabel=V_NPR.INNMATEHAST-K_NPR.EPISODE.

8. Helsedataservice [Health Data Service]. Omsorgsnivå [Service level]. [Internet]. 2025 [cited 2025 May 13]. Available from: https://helsedata.no/no/variabler/?page=search&variabel=V_NPR.OMSORGSNIVA-K_NPR.EPISODE.

9. Tveit OG, Ruud T, Hanssen-Bauer K, Haavet OR, Hussain A. An explorative study of factors associated with treatment in specialized mental health care centers among GP patients in Norway. BMC Health Serv Res. 2021;21(1):960. https://doi.org/10.1186/s12913-021-06982-4

10. Pruckner GJ, Stiftinger F, Zocher K. When women take over: Physician gender and health care provision. J Health Econ. 2025;102:103000. https://doi.org/10.1016/j.jhealeco.2025.103000
