# Supplementary material for: The effect of general practitioners’ sex and age on patients’ healthcare utilization: a Norwegian registry study
Source: Res Health Serv Reg. 2026 Feb 6;5:4. doi: 10.1007/s43999-026-00086-4 (PMC12881246; doi:10.1007/s43999-026-00086-4)
Supplement: Supplementary file 2 — Supplementary Material 2 [file 43999_2026_86_MOESM2_ESM.pdf]

Supplementary Results

Bennett SM*, Anthun KS, Bjerkeset O, Godager G, Bjørngaard JH, Edwards CH. *The effect of general practitioners' sex and age on patients' healthcare utilization: a Norwegian registry study.* Research in Health Services & Regions.

***Corresponding author:**
Schyler Marie Bennett | schyler.bennett@ntnu.no | Department of Public Health and Nursing, Faculty of Medicine and Health Sciences, NTNU – Norwegian University of Science and Technology; Trondheim, Norway

General notes for Supplementary Figures

Exposure variables (shapes)

- GP sex: **circles**
  - Fig. S3, sex-stratified: **circles** = female patients; **diamonds** = male patients
- GP age: **triangles**
  - Fig. S4, sex-stratified: **triangles** = female patients; **squares** = male patients

Outcome representation

- Mental healthcare services are represented by **filled symbols**.
- Somatic healthcare services are represented by **hollow symbols**.
- Healthcare service types (**acute inpatient, non-acute inpatient, non-acute outpatient, out-of-hours**) are shown in separate **rows**.
- **Left panels** show the relative risk of having a contact.
- **Right panels** show the change in the number of contact days per year.
- **Horizontal lines** indicate **95% confidence intervals**.

Patient groups (colors)

Mental health diagnosis history (Figs. S1–S7)

- **Black**: all patients
- **Light blue**: diagnosis 1 year before assignment
- **Purple**: diagnosis 2 years before assignment
- **Pink**: diagnosis 3 years before assignment
- **Turquoise**: recurrent mental health problem

A mental health diagnosis is defined as having a P-diagnosis from International Classification of Primary Care 2 (ICPC-2) ***and/or*** *an F-diagnosis from International Classification of Diseases Version 10 (ICD-10). The 2 years and 1 year before groups include only* ***new*** *diagnoses (i.e. excluding those with a diagnosis 3 years before and 2 or 3 years before, respectively). A recurrent mental health problem is defined as having a P- and/or F-diagnosis in all three years before assignment.*

Switching after assignment (post-assignment GP-switching analysis; Figs. S8–S9)

- **Dark green**: no further switches
- **Light blue**: patient-initiated switch in year 1 after assignment
- **Light purple**: patient-initiated switch in year 2 after assignment
- **Light pink**: patient-initiated switch in year 3 after assignment

- **Dark orange**: second assignment due to GP closure or reduction of list any time in the three years following the first assignment

Years relative to assignment (line styles)

- **Long dashed**: 2 years before assignment
- **Dot-dashed**: 1 year before assignment
- **Dashed**: 1 year after assignment
- **Solid**: 2 years after assignment
- **Two-dashed**: 3 years after assignment

Figs. S3 and S4 show only the results for the second year after assignment, as noted in the individual captions.

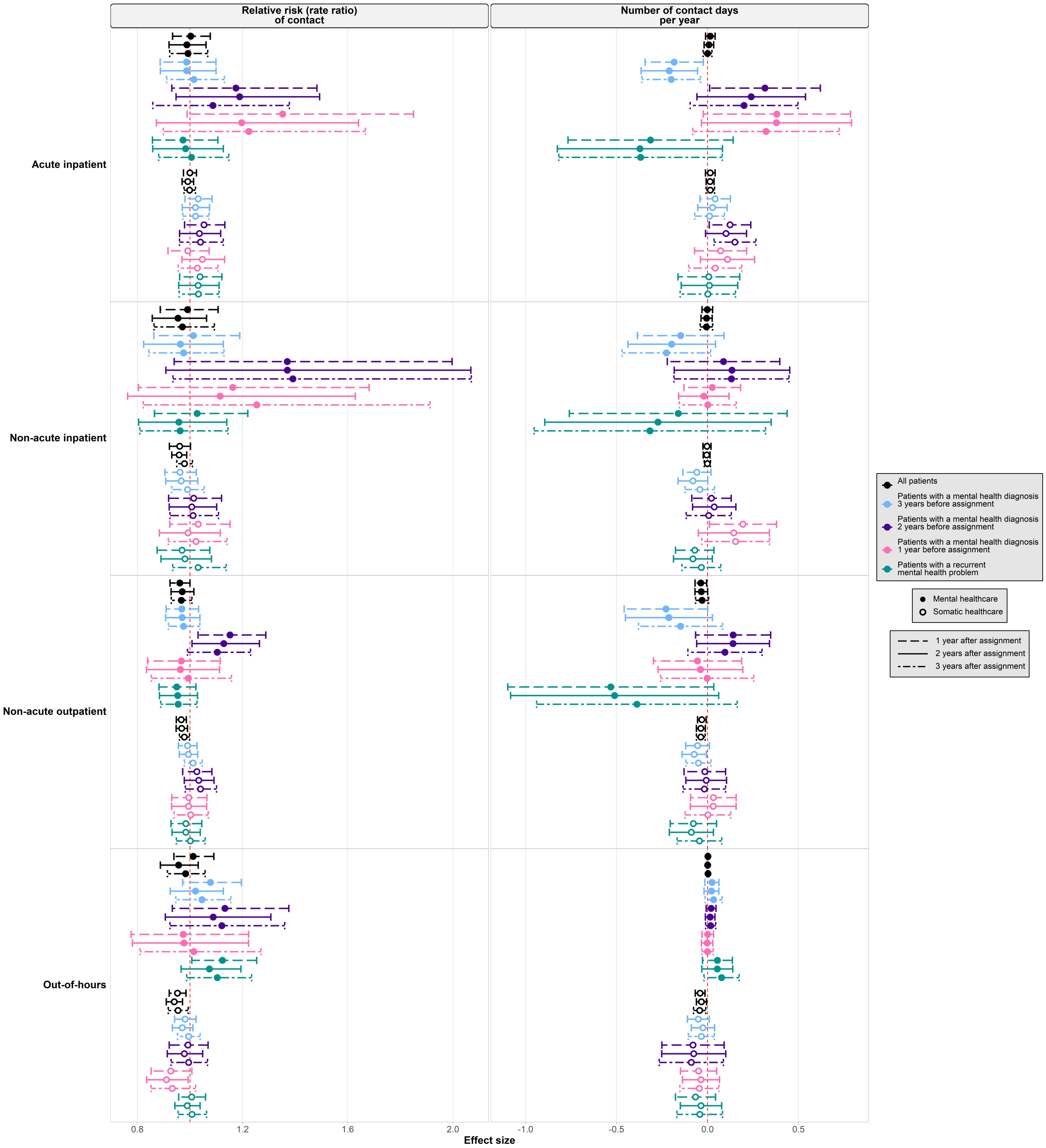

***Fig. S1** Estimated association of GP sex with healthcare utilization, by year after assignment and patient mental health history.*

*Notes: The figure shows the estimated association of **assignment to a male general practitioner (GP)** (female GP as reference category) with the **relative risk of contact** (left panel) and **number of contact days per year** (right panel) with specialist and out-of-hours healthcare. See General Notes for further definitions of patient groups, line styles, and symbols*

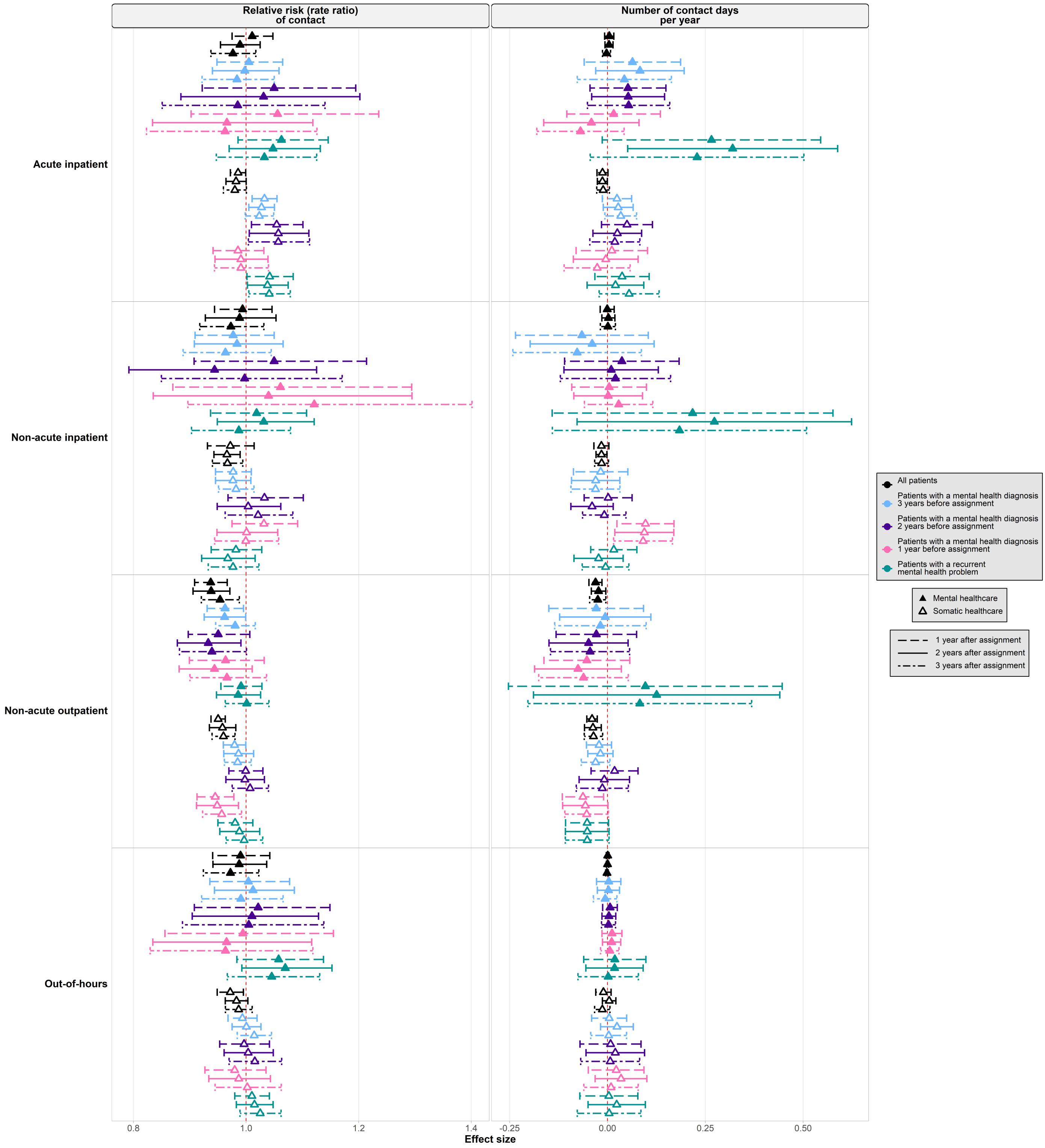

***Fig. S2** Estimated association of GP age with healthcare utilization, by year after assignment and patient mental health history.*

*Note: The figure shows the estimated association of **each 10-year increase in general practitioner (GP) age** with the **relative risk of contact** (left panel) and **number of contact days per year** (right panel) with specialist and out-of-hours healthcare. See General Notes for further definitions of patient groups, line styles, and symbols*

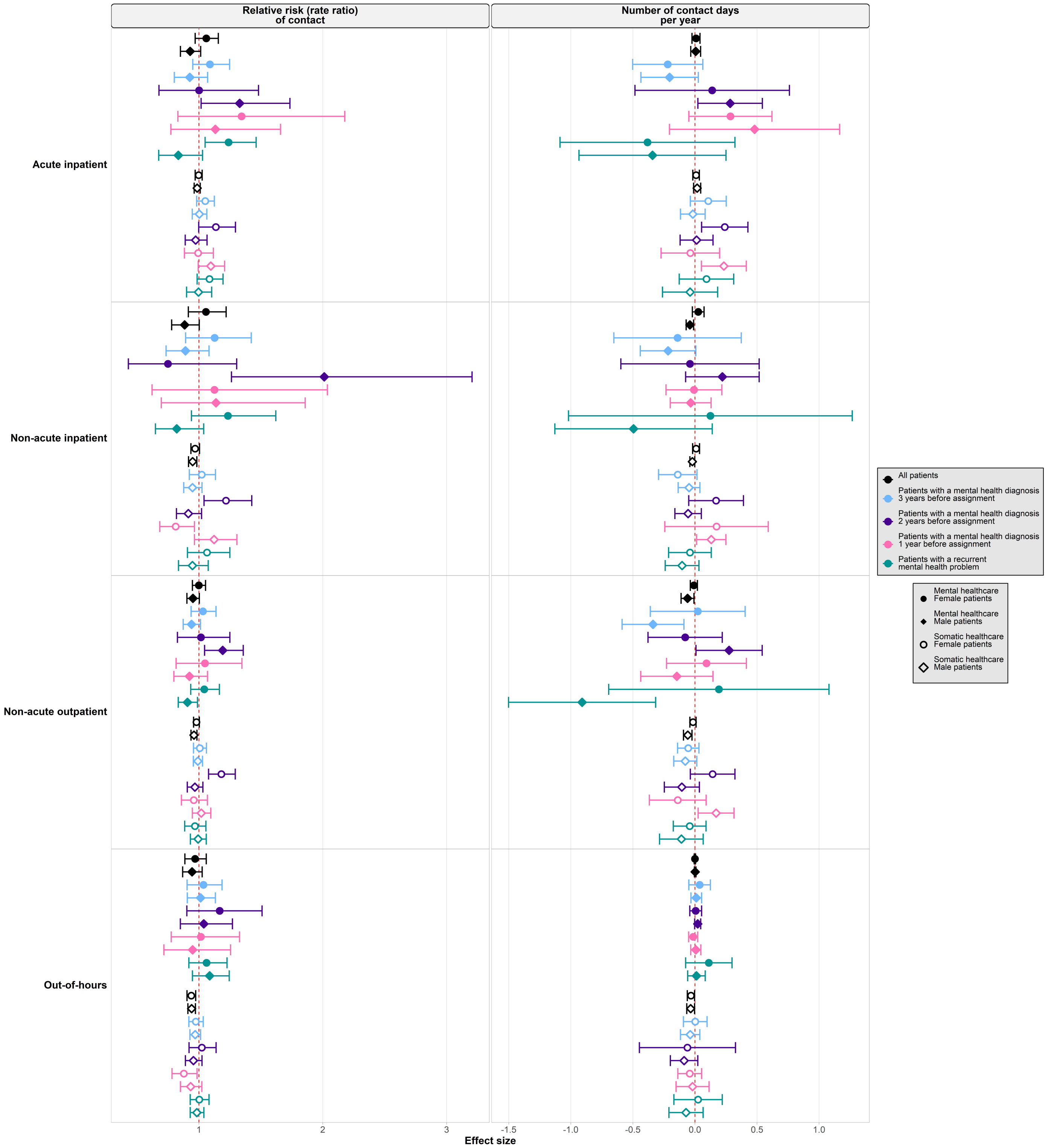

Fig. S3 *Estimated association of GP sex with healthcare utilization, by patient sex.*

Note: The figure shows the estimated association of ***assignment to a male general practitioner (GP)*** *(female GP as reference category) with the* ***relative risk of contact*** *(left panel) and* ***number of contact days per year*** *(right panel) with specialist and out-of-hours healthcare,* ***stratified by patient sex****.* ***Circles*** *represent female patients, and* ***diamonds*** *represent male patients. All estimates are from the second year after assignment. See General Notes for further definitions of patient groups, line styles, and symbols*

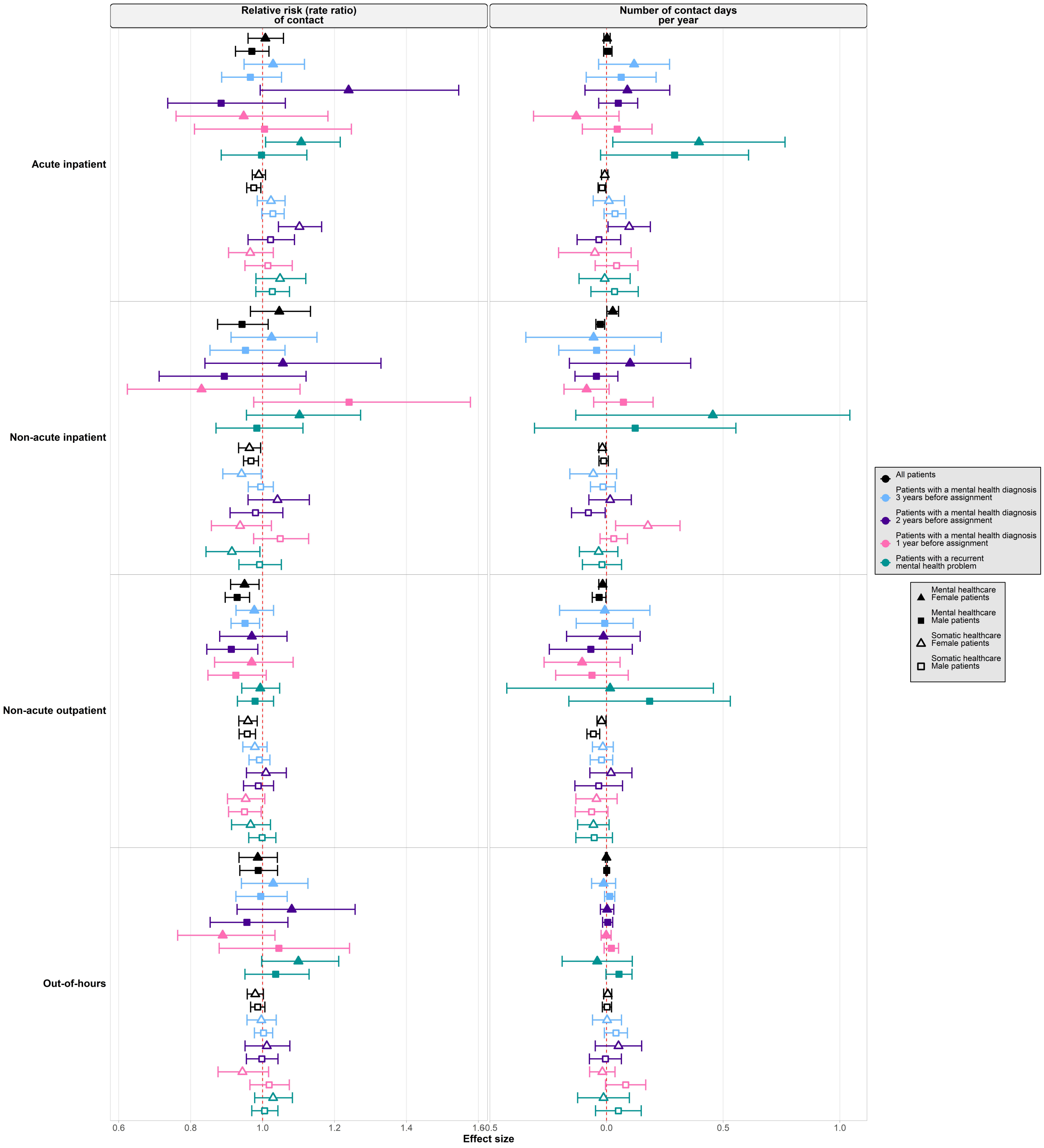

Fig. S4 *Estimated association of GP age with healthcare utilization, by patient sex.*

*Note: The figure shows the estimated association of **each 10-year increase in general practitioner (GP) age** with the **relative risk of contact** (left panel) and **number of contact days per year** (right panel) with specialist and out-of-hours healthcare, **stratified by patient sex**. **Triangles** represent female patients, and **squares** represent male patients. All estimates are from the second year after assignment. See General Notes for further definitions of patient groups, line styles, and symbols*

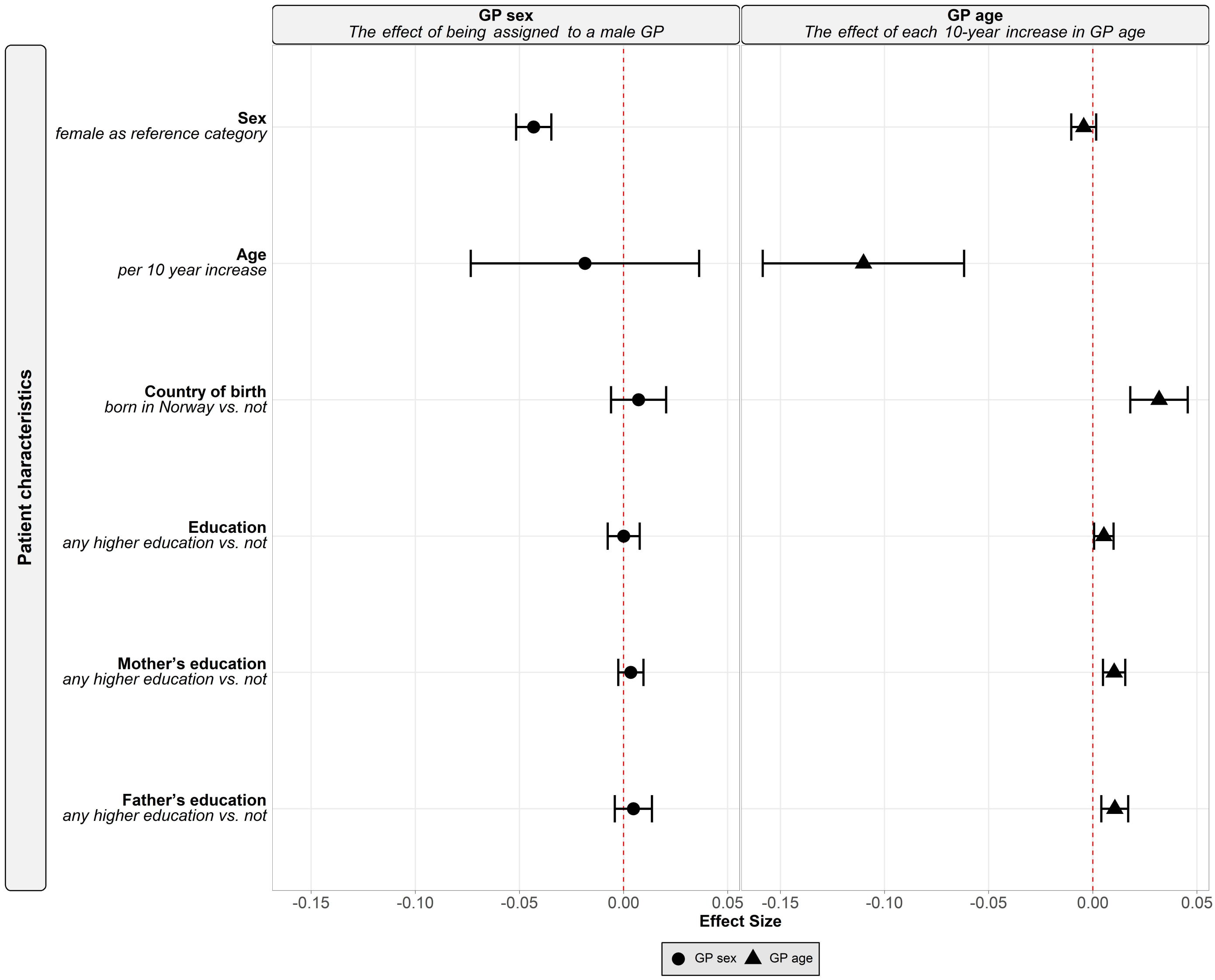

***Fig. S5** Assessment of the assignment process: balance test on the association of GP characteristics with patient characteristics.*

*Note: The figure shows the association of **assignment to a male general practitioner (GP)** (left panel, circles) or **each 10-year increase in GP age** (right panel, triangles) with patient characteristics. Patient characteristics include **sex** (male or female, female as reference group), **age** (continuous in years, scaled for each 10-year increase [divided by 10]), **country of birth** (born in Norway to Norwegian or foreign parents, or born outside of Norway to Norwegian or foreign parents), **education level** (yes/no any education above high school level achieved in October of year of assignment), and **father's** and **mother's education level** (yes/no any education above high school level in the year patient turned 16 years old)*

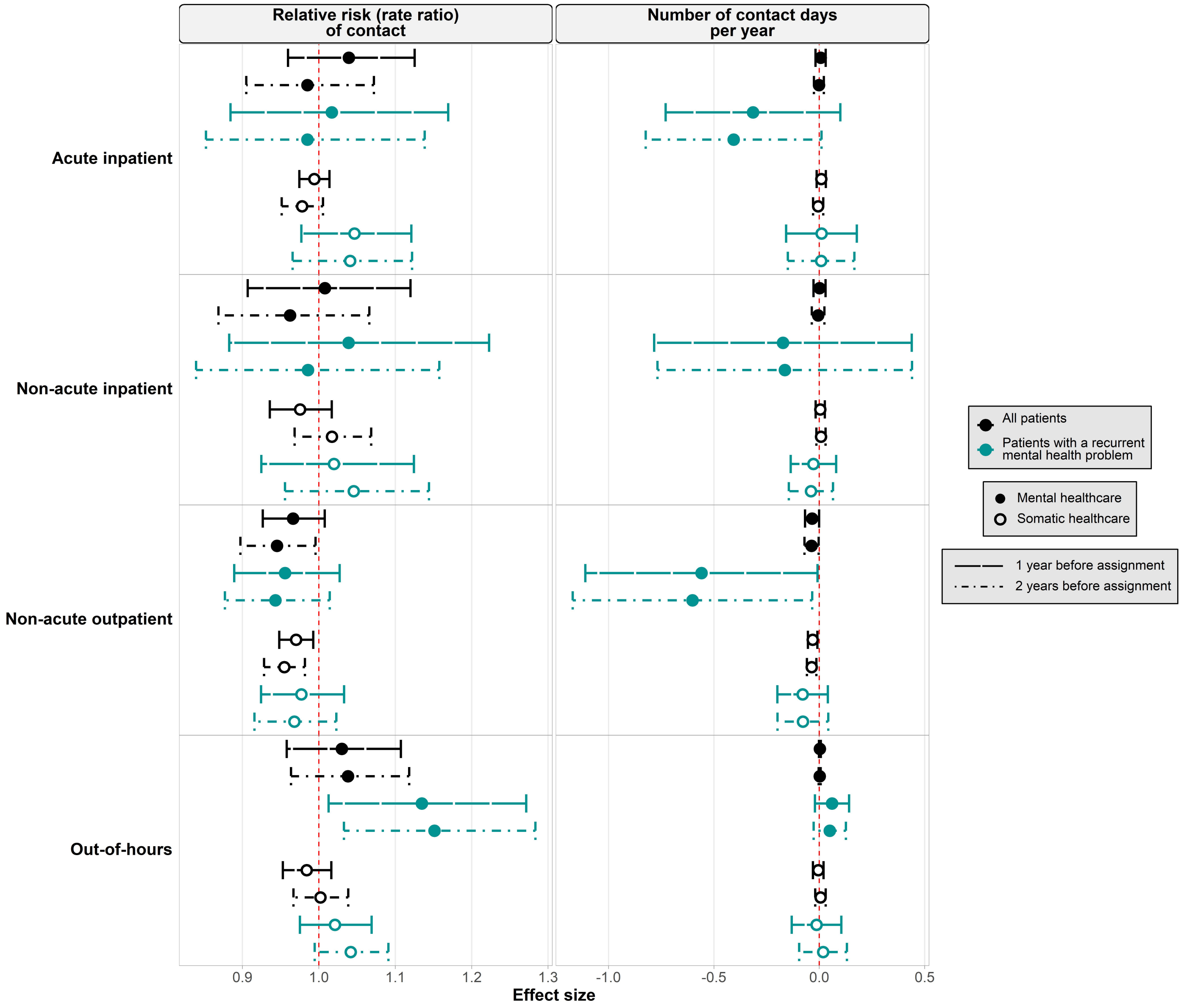

Fig. S6 *Estimated association of GP sex with healthcare utilization prior to assignment.*

Note: The figure shows the estimated association of ***assignment to a male general practitioner (GP)*** *(assignment to a female GP as the reference category) with the* ***relative risk of contact*** *(left panel) and the* ***number of contact days per year*** *(right panel) with specialist and out-of-hours healthcare* ***prior to assignment****. See General Notes for further definitions of patient groups, line styles, and symbols*

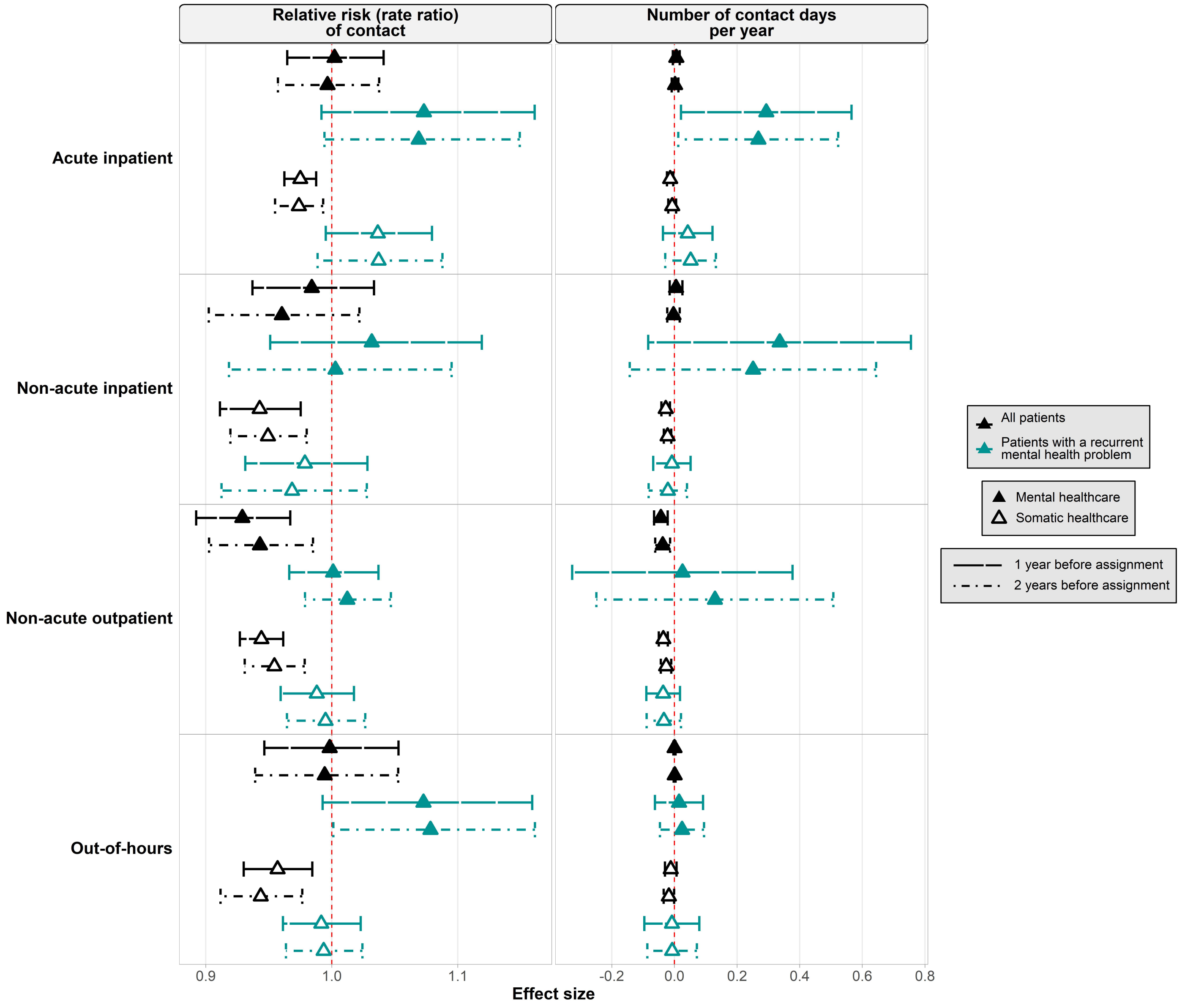

Fig. S7 *Estimated association of GP age with healthcare utilization prior to assignment.*

*Note: The figure shows the estimated association of **each 10-year increase in general practitioner (GP) age** with the **relative risk of contact** (left panel) and the **number of contact days per year** (right panel) of contact with specialist and out-of-hours healthcare **prior to assignment**. See General Notes for further definitions of patient groups, line styles, and symbols*

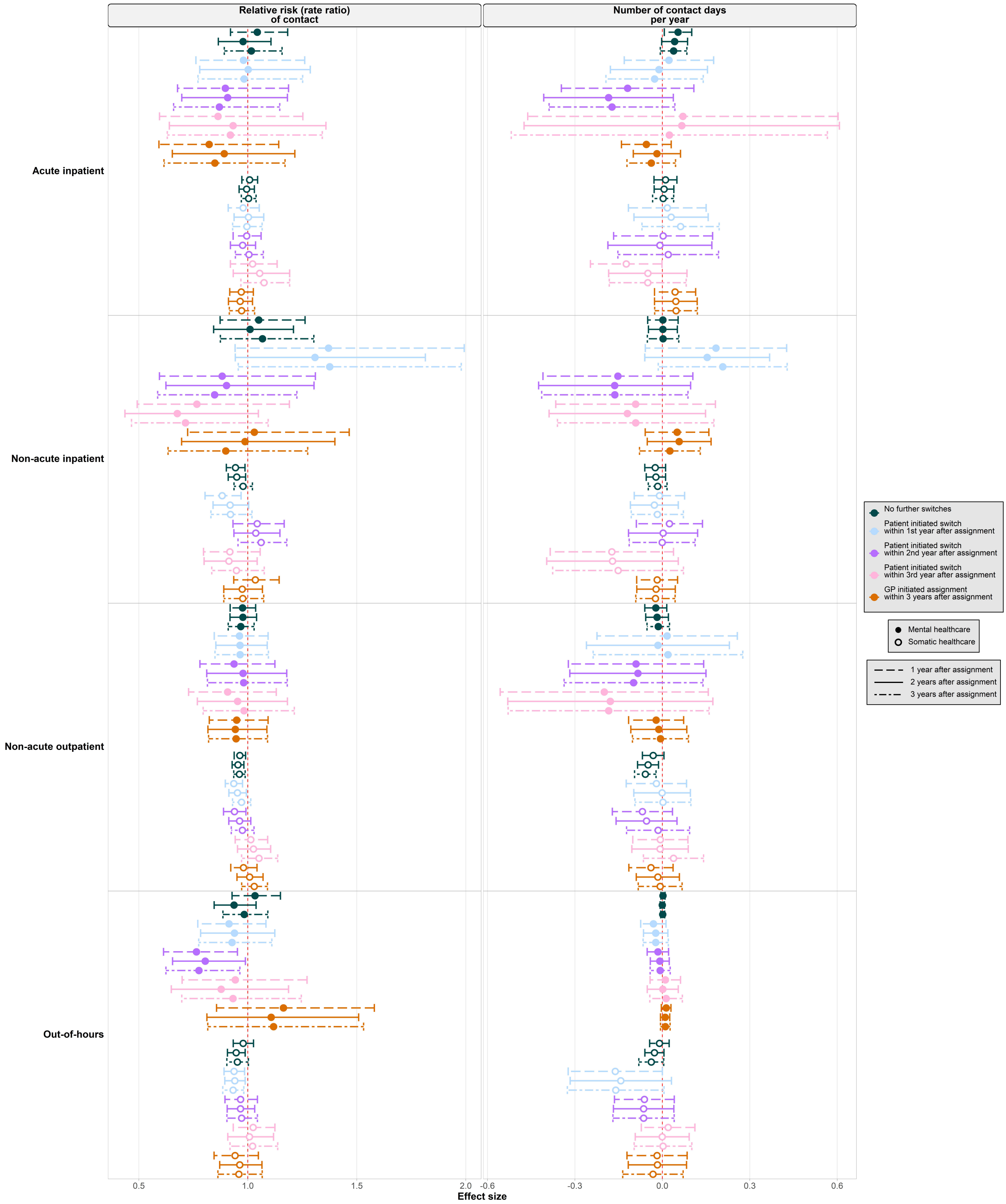

***Fig. S8** Post-assignment GP switching: Estimated association of GP sex with healthcare utilization, by year after assignment and switches after assignment.*

*Note: The figure shows the estimated association of **assignment to a male general practitioner (GP)** (assignment to female GP as the reference category) with the **relative risk of contact** (left panel) of and the **number of contact days per year** (right panel) with specialist and out-of-hours healthcare amongst patients who switched GPs after assignment. See General Notes for further definitions of patient groups, line styles, and symbols*

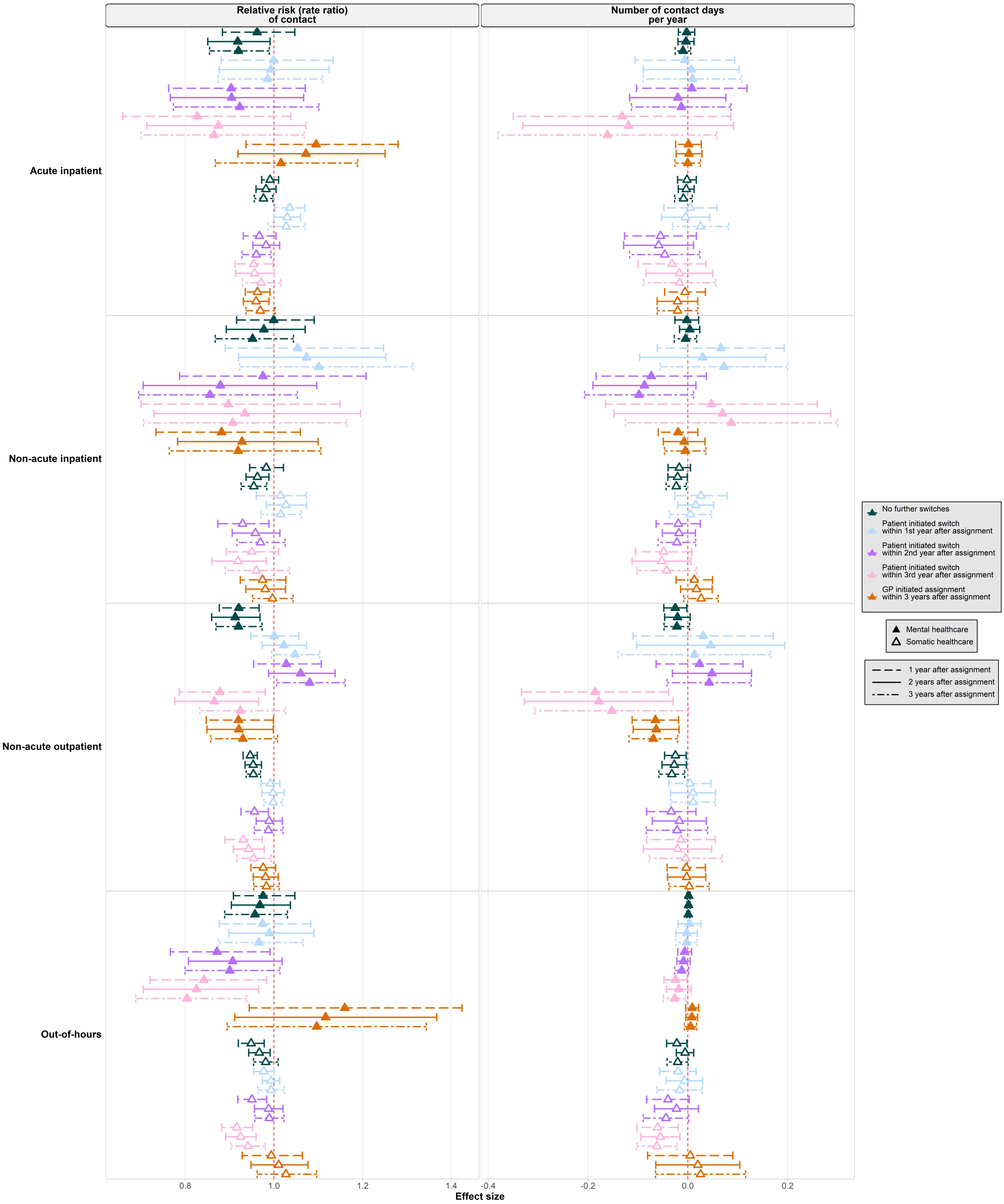

***Fig. S9** Post-assignment GP switching: Estimated association of GP age with healthcare utilization, by year after assignment and switches after assignment.*

*Note: The figure shows the estimated association of **each 10-year increase in general practitioner (GP) age** with the **relative risk of contact** (left panel) of and the **number of contact days per year** (right panel) of contact with specialist and out-of-hours healthcare, stratified by patients who switched GPs after assignment. See General Notes for further definitions of patient groups, line styles, and symbols*

Table S1 *Hypothetical scenario to illustrate system-level impacts of findings.*

		Patients assigned to a GP with contact in 2019	Patients assigned to a female GP with contact in 2019	Estimated relative risk (RR) of assignment to a male GP		Additional patients who would have had contact in 2019 if assigned to a male GP			
Type of care		*N*	*N*	*RR*	*95% CI*	*N* [a]	*95% CI*	*% change* [b]	*95% CI (%)*
Mental	Acute inpatient	14,350	6,518	0.99	(0.92 – 1.06)	6,441	(5,999 – 6,917)	-0.53 %	(-3.62 to 2.78)
	Non-acute inpatient	5,362	2,472	0.95	(0.86 – 1.06)	2,359	(2,118 – 2,628)	-2.11 %	(-6.61 to 2.91)
	Non-acute outpatient	72,438	34,479	0.97	(0.93 – 1.01)	33,463	(32,005 – 34,988)	-1.40 %	(-3.41 to 0.70)
	Out-of-hours	22,371	9,904	0.96	(0.89 – 1.03)	9,476	(8,790 – 10,215)	-1.91 %	(-4.98 to 1.39)
Somatic	Acute inpatient	256,580	116,655	0.99	(0.97 – 1.01)	115,681	(113,140 – 118,279)	-0.38 %	(-1.37 to 0.63)
	Non-acute inpatient	158,517	72,595	0.96	(0.93 – 0.99)	69,607	(67,575 – 71,699)	-1.89 %	(-3.17 to -0.57)
	Non-acute outpatient	600,430	276,042	0.97	(0.95 – 0.99)	267,409	(261,525 – 273,426)	-1.44 %	(-2.42 to -0.44)
	Out-of-hours	345,929	157,256	0.94	(0.91 – 0.97)	147,869	(143,089 – 152,808)	-2.71 %	(-4.10 to -1.29)

a. Calculated by multiplying the number of patients assigned to a female GP with contact in 2019 with the relative risk of being assigned to a male GP.

b. Calculated by subtracting the number of patients assigned to a female GP with contact in 2019 from the number of additional patients who would have had contact in 2019 if they had been assigned to a male GP, and then dividing this value by the number of patients assigned to a GP with contact in 2019.
